# Circulating Tumor Cells: Clinically Relevant Molecular Access Based on a Novel CTC Flow Cell

**DOI:** 10.1371/journal.pone.0086717

**Published:** 2014-01-29

**Authors:** Jessamine P. Winer-Jones, Behrad Vahidi, Norma Arquilevich, Cong Fang, Samuel Ferguson, Darren Harkins, Cory Hill, Erich Klem, Paul C. Pagano, Chrissy Peasley, Juan Romero, Robert Shartle, Robert C. Vasko, William M. Strauss, Paul W. Dempsey

**Affiliations:** 1 Research and Development, Cynvenio Biosystems, Westlake Village, California, United States of America; 2 Engineering, Cynvenio Biosystems, Westlake Village, California, United States of America; University of Kentucky College of Medicine, United States of America

## Abstract

**Background:**

Contemporary cancer diagnostics are becoming increasing reliant upon sophisticated new molecular methods for analyzing genetic information. Limiting the scope of these new technologies is the lack of adequate solid tumor tissue samples. Patients may present with tumors that are not accessible to biopsy or adequate for longitudinal monitoring. One attractive alternate source is cancer cells in the peripheral blood. These rare circulating tumor cells (CTC) require enrichment and isolation before molecular analysis can be performed. Current CTC platforms lack either the throughput or reliability to use in a clinical setting or they provide CTC samples at purities that restrict molecular access by limiting the molecular tools available.

**Methodology/Principal Findings:**

Recent advances in magetophoresis and microfluidics have been employed to produce an automated platform called LiquidBiopsy®. This platform uses high throughput sheath flow microfluidics for the positive selection of CTC populations. Furthermore the platform quantitatively isolates cells useful for molecular methods such as detection of mutations. CTC recovery was characterized and validated with an accuracy (<20% error) and a precision (CV<25%) down to at least 9 CTC/ml. Using anti-EpCAM antibodies as the capture agent, the platform recovers 78% of MCF7 cells within the linear range. Non specific recovery of background cells is independent of target cell density and averages 55 cells/mL. 10% purity can be achieved with as low as 6 CTCs/mL and better than 1% purity can be achieved with 1 CTC/mL.

**Conclusions/Significance:**

The LiquidBiopsy platform is an automated validated platform that provides high throughput molecular access to the CTC population. It can be validated and integrated into the lab flow enabling CTC enumeration as well as recovery of consistently high purity samples for molecular analysis such as quantitative PCR and Next Generation Sequencing. This tool opens the way for clinically relevant genetic profiling of CTCs.

## Introduction

Cancer metastasis involves the dissemination of primary tumor cells through the bloodstream and lymphatics. In cancer patients, rare cells have been observed, recovered and described as circulating tumor cells (CTC) [1], [2]. The implicit relationship between cancer metastasis and CTCs has long been postulated [3]; however, the specific identity of the cells found in the circulation of cancer patients and normal healthy volunteers has been clouded by assumptions and technical limitations [4]. With recent technical advances, it has become possible to develop molecular descriptions of circulating tumor cells [5], [6]. Thus it is finally possible to progress from the classic phenotypic/morphological descriptions of rare cells found in the circulatory system, and propose descriptions, or classifications, that are based upon modern molecular biology.

Epithelial derived cancers account for 80–90% of malignancies, and it has been observed that CTCs found in patients with solid epithelial tumors express epithelial markers such as the epithelial cell adhesion molecule (EpCAM) and cytokeratin (CK). Over the last 25 years, a series of tools have been developed that focus on recovery of rare cells expressing these epithelial characteristics [2], [7], [8]. Through the optimization of capture methods based on this phenotype, it has been shown that elevated numbers of CTCs can be detected in the blood of patients with cancer [7], [9], [10]. One overlooked aspect of this processing is that each different method applies a technology-related biased definition of the CTC. These subjective definitions have consequences for the enumerative, phenotypic, or in some cases prognostic association with the disease state [11].

The evolution of our understanding of the circulating tumor cell from a phenomenology to a clinical analyte requires precise delineation of the relationship between a rare cell in circulation and the tumor from which it is derived. To make these delineations, a series of changes in technical capability and biological understanding are required. From the biologic perspective, blood is a complex tissue that contains many different nonhematologic populations [12], [13]. The identification and derivation of all these nonhematologic cells in circulation is incomplete and may include noncancer derived epithelial cells [14]. Therefore the technical implications of sampling nonhematologic cells from blood must be understood in the context of this biological complexity. Further compounding this analysis is the complexity of cells observed in the disease states termed “cancer”. The molecular pathology of cancer has been expanded from the simplistic view of a few driver mutations to the heterogenous population with a complex array of somatically acquired mutations. Thus, using mutations to genetically define lineage within a tumor population by methods like Next Generation Sequencing (NGS) lead to the understanding that the simple, tree-based phylogenies present an oversimplification of the natural history of tumor evolution [15]. As a consequence, any molecular analysis of CTCs must reflect these different sources of complexity [6]. To overlook this complexity is to miss the actual significance of CTCs for understanding tumor progression and metastasis.

This paper describes the validation of a high throughput CTC flow cell and the associated automated LiquidBiopsy platform and protocols that enable the rapid and robust recovery of CTCs in a manner that allows direct molecular analysis. The platform allows recovery of target populations with sufficient purity to enable facile molecular analysis with a variety of tools. Molecular analysis has the capacity transform CTC analysis from a simple diagnostic triaging tool into an access point for genetic material which can drive appropriate treatment selection based on molecular profiling.

## Materials and Methods

### Ethics Statement

The healthy control blood samples used in this report were obtained from Hemacare Corp (Van Nuys, CA). Subjects provided written informed consent under an IRB approved by the Biomedical Research Institute of America (San Diego, CA).

### Study Subjects

Acceptable subjects had never received a diagnosis of cancer and were currently healthy. The blood samples had a quantity of a minimum of 8 mL and showed no signs of hemolysis. Blood was run on the LiquidBiopsy platform within 96 hours of blood draw.

### Flow Cell Fabrication

All fluidic geometries were created using class A stamping tools in an electromagnetic press. The layers were made from a hydrophilic polyester film with a polyester membrane switch spacer adhesive. The layers are then stacked and aligned using registration holes and laminated to create the microfluidic pathways. The polyester is optically transparent with minimum autofluorescence. Each flow cell is laminated to a glass slide and then inspected for correct slide placement. For manual runs, a female luer port (Valve Plastic, Fort Collins, CO) was mounted to the sample port and barb ports (Valve Plastic, Fort Collins, CO) were mounted over the top buffer, bottom buffer and outlet ports.

### Sample Processing

Unless otherwise stated, all reagents and materials were purchased from Fisher Scientific (Waltham, MA). Blood was drawn into 10 mL K_2_EDTA vacutainers (BD, Franklin Lakes, NJ) then fixed with the LiquidBiopsy fixative (Cynvenio Biosystems, Westlake Village, CA) within 4 hours of collection. Blood was stored at room temperature and processed within 96 hours of fixation. For cell culture spiked experiments, fixed MCF7, HCC1419, H1650, BT20 or SK-Mel-28 cells (ATCC, Manassas, VA) were added to the control blood. After fixation the blood was brought to a volume of 50 mL with wash buffer (1× PBS, 10% FBS and 0.02M EDTA) and spun down at 500× g for 15 min at 4°C. The blood was washed and resuspended to the original blood volume with wash buffer. Then, 8 mL of the washed blood was transferred to a 15 mL conical and 160 µL FcR Block antibody (Miltenyi, Auburn, CA) was added. The sample was incubated on ice for 15 minutes.

Ferrofluid was prepared by adding iMAG streptavidin beads (BD Biosciences, Auburn, CA) to titrated amounts of biotinylated anti-EpCAM (Santa Cruz Biotechnology, Santa Cruz, CA), anti-Her2 (Santa Cruz Biotechnology, Santa Cruz, CA), anti-MelCAM (Santa Cruz Biotechnology, Santa Cruz, CA), anti-Muc1 (BD, Franklin Lakes, NJ) or anti-Trop2 (BD, Franklin Lakes, NJ) in a solution of 10% FBS in EMEM. This antibody ferrofluid solution was incubated for 30 minutes on ice and then washed twice with CMEM before transferring into the blood sample. After mixing, the tube was placed in a BD iMAG magnet (BD, Franklin Lakes, NJ) on ice for 30 minutes. At intervals, the sample was agitated to resuspend the beads and cells. The samples received a final wash with 50 mL of wash buffer. The sample was returned to it's original volume using wash buffer and stored at 4°C for up to 4 hours prior to processing on the CTC flow cell.

For manual CTC flow cell runs, spiked cells were detected either by labeling with CFSE (Life Technologies, Grand Island, NY) or FITC conjugated CAM5.2 anti-CK antibody (BD, Franklin Lakes, NJ). The flow cell was operated by delivering buffers using Milligat pumps (GlobalFIA, Fox Island, WA). Captured cells were manually counted in the flow cell using an Eclipse E80i fluorescent microscope (Nikon Instruments, Melville, NY). Target cells previously labelled with CFSE or FITC anti-CK were enumerated by manual inspection. All other 4′,6-diamidino-2-phenylindole (DAPI) positive events, regardless of the CD45 status, were counted as background.

The automated LiquidBiopsy platform was designed to continuously flow liquid through a laminar flow cell using PLC controlled pumps and a pipetting arm. The three layer flow was initialized with PBS in the top and middle layers and density controlled Iohexol buffer on the bottom (Accurate Chemical, Westbury, NY). Once sheath flow was established the middle layer was switched to labeled blood sample and loaded at 5 mL/hr. Following the loading of the blood, the device was washed with PBS and the top buffer turned off. Subsequently, a 7.5 minute permeabilization step using PBS with 0.5% Triton X-100 (PBS/Triton) flowing at a rate of 8 mL/hr was initiated. Once permeabilized, the cells were labeled in flow conditions for 7.5 minutes with DAPI, FITC conjugated pan-cytokeratin antibody and APC conjugated anti-CD45 antibody (Santa Cruz Biotechnology, Santa Cruz, CA) in PBS/Triton. Cells were washed again with PBS and then labeled for 7.5 minutes with AlexaFluor-488 anti-FITC (InVitrogen, Carlsbad, CA) in PBS/Triton. After a final wash, the flow chamber was filled with a stabilization buffer (PBS, 2% FBS and 0.5% Triton).

### Sample Evaluation

After capture on the automated platform, the CTC flow cell ports are covered and the flow cell was inverted so all cells remained on the glass surface. The entire capture region of the flow cell was scanned and analyzed using a Leica DM4000 microscope and the Ariol software (Leica, Buffalo Grove, IL). DAPI events were identified by the Ariol software and sorted into 4 categories (CK+/CD45−, CK−/CD45+, CK+/CD45+, CK−/CD45− or debris) curated by a human operator. Sorting was achieved by first identifying the population of CK−/CD45+ mononuclear white blood cells (WBC). The WBC population was used to set the threshold for CK positivity (2 standard deviations above the mean WBC staining for CK). Cells were determined to be CTC (CK+/CD45−) if they had a FITC signal above the threshold and no CD45 signal. CK+/CD45+ cells had a FITC signal above the threshold and visible CD45 staining. CK−/CD45− cells had a FITC signal below the threshold and no CD45 staining.

### Spin Elution

After imaging, the cells were recovered from the flow cell into a PCR tube using the custom SpinElute tube (Cynvenio Biosystems, Westlake Village, CA). Cells were recovered from the flow cell by sonication in a petri dish to release the cells from the cover slip. The flow cell was placed into the SpinElute Tube with a 0.5 mL microcentrifuge tube attached (Figure S5A) and centrifuged at 100 g for 1 minute. The flow cell was washed once and cells were recovered in the same way. Recovered cells were pelleted by centrifugation at 5000 g for 10 minutes. The PCR tube is placed in a magnetic holder and the supernatent removed leaving 20 µL of volume at the bottom of the tube. The cell pellet was frozen and stored at −80°C.

Elution efficiency was assessed by imaging and PCR. The elution efficiency of releasing captured cells from the flow cell was evaluated by spiking 4 normal healthy donor (NHD) blood samples with 30 MCF7 cells/mL and processing them using the LiquidBiopsy platform. The flow cells were imaged before and after elution and the MCF7 target cells and donor derived cells were enumerated. To assess the ability to recover all the genomic DNA from fixed cells which had been eluted from a flow cell, 500 fixed WBCs were either added directly to a PCR tube or injected into and recovered from a flow cell. The cell pellet was processed by proteinase K digestion using our standard protocol described in the molecular analysis section. The quantity of gDNA released was assessed in triplicate using a TaqMan probe for Chromosome 9p.

### Validation Study Design

In the intra-assay test, a set of four samples from the same donor were spiked with either 9 or 90 MCF7 cells/mL as indicated. Each set of four samples was processed on the same day, by the same operator, on the same platform. In the inter-assay test, variability was assessed by having two operators prepare duplicate samples at two concentrations (9 and 90 cells/mL) and then running one sample of each pair on separate platforms as shown in Table S1. This study was repeated over 5 days. 4 donors were used each day and each donor gave two blood samples which were split between operators to minimize effects of normal donor variation. Two different operators and two different platforms were used during the course of this study.

### Imaging of Laminar Flow

Laminar flow within the flow cell was visualized using a top buffer of PBS containing green fluorescent dye (Bright Dyes, Miamisburg, OH), a sample buffer of PBS containing red fluorescent dye (Bright Dyes, Miamisburg, OH) and a density adjusted bottom layer of 50% sucrose in PBS containing the green fluorescent dye. Imaging was done with an Olympus Fluoview 1000 MPE (upright microscope) and an Olympus Fluoview 500 (inverted microscope) to image from the top and bottom respectively.

### Molecular Analysis

Cell pellets for molecular analysis were prepared by adding 90, 30, 9 or 0 cells/mL to whole blood. A 0.2 mL aliquot of unprocessed blood was reserved and red blood cells (RBC) were removed with VersaLyse (Beckman Coulter, Brea, CA). Alternatively, the samples were processed on the LiquidBiopsy platform and the cell pellet was recovered using the Cynvenio SpinElute tube (Cynvenio, Westlake Village, CA). Cell pellets were digested with 5 µl of Digest Buffer (Cynvenio, Westlake Village, CA) containing 2 mg/mL proteinase K for 4 hours. WBC control pellets (both spiked and unspiked) were treated identically but 50 µl of Digest Buffer was added. Then, 2.5 ng of WBC DNA (as determined by NanoDrop) or the SpinElute cell pellet digests were amplified with the GenomiPhi WGA kit (GE Healthcare Biosciences, Pittsburgh, PA) according to the manufacturer's instructions. Resulting DNA was cleaned with a 0.5X ratio of Ampure XP reagent (Beckman Coulter, Brea, CA), washed twice with 70% ethanol and eluted in TE (Life Technologies, Carlsbad CA). Finally, 50 ng of DNA from each sample was used as template for PCR reaction using EGFR_6223_mu and EGFR_reference mutation detection assays with TaqMan Genotyping Master Mix according to manufacturer's instructions. The reactions were visualized on a ViiA7 Real Time PCR instrument (Applied Biosystems/Life Technologies, Grand Island, NY). Change in threshold cycle (∂Ct) was calculated for each template by subtracting the threshold cycle (Ct) of the reference probe from the mutant probe.

### Statistical Methods

Data from a normal distribution, such as recovery of a spiked sample, are reported as mean ± standard deviation. Data from a non-normal distribution, such as recovery of nontarget cells from normal donor controls, are reported as median. Normality was determined by use of a Q-Q plot. The linear regression analysis was performed in Excel (Microsoft, WA). The factorial ANOVA was performed using MacANOVA software (University of Minnesota, MN) using the model Recovery = Constant+Day+Operator+Platform+Operator*Platform and the alpha level 0.05. The equations for percent error and coefficient of variation are as follows: percent error = abs(measured value - expected value)/expected value * 100 and coefficient of variation = standard deviation/mean * 100.

## Results

### CTC Flow Cell Design and Function

**Figure 1:**
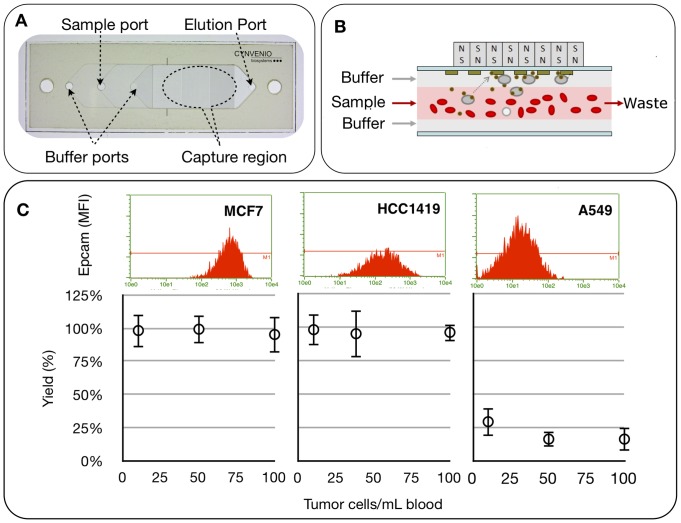
CTC Flow cell operation and performance. (A) The LiquidBiopsy CTC flow cell. (B) A cartoon illustrating how target cells are pulled from the sheath flow while non targets move through the flow cell unhindered. (C) Efficiency of recovery of target cells using EpCAM based recovery: Incremental numbers of MCF7 (N = 34), HCC1419 (N = 27) or A549 (N = 65) cells were spiked into NHD blood and purified on the CTC flow cell. Recovered cells were enumerated in the flow cell. Graphs show number of targets spiked per mL of blood against the yield +/−1 SD from an average of between 7 and 27 experiments.

Recovery of CTC on the LiquidBiopsy platform relies on fluid phase labeling of whole blood with a functionalized ferrofluid followed by high throughput partitioning of CTC from blood using a CTC sheath flow cell. Efficient isolation of rare target cells (such as labeled CTCs) to a high purity requires a device that can: 1) minimize handling steps such as depletion or red blood cell elimination, 2) maximize recovery of labeled target cells at a high throughput, 3) minimize nonspecific binding of non target cells to device surfaces, 4) eliminate sample transfer loss by integrating separation and classification in the same chamber and 5) provide easy recovery of these small numbers of cells for additional analysis. To these ends, the CTC flow cell was designed with a form factor similar to a standard pathology slide supporting standard imaging platform compatibility (Figure 1A). It uses a multilayer sheath flow with density adjusted buffers to prevent nonspecific binding of non target cells to chamber surfaces (Figure 1B).

Density adjusted buffers isolate the sample in a sheath flow. This serves to minimize non target interactions with the device surfaces (Figure S1 and S2). Labeled blood cells enter through the sample port, flows through the capture region and exits the elution port without coming in contact with the surfaces of the capture region (Figure 1B). In contrast, immunomagnetically labeled target cells are magnetophoretically lifted from the sample layer, through the top buffer layer and captured on the upper glass surface by the large magnetic field gradients (Figure 1B and S3).

Magnetophoretic mobility of a labelled population can be altered by 1) changing the antibody binding capacity, 2) changing the magnetic moment of the capture particles or 3) incrementing the magnetic flux density within the device [16]. Nanometer-sized iron particle were selected because such paramagnetic ferrofluids better approximate fluid phase labeling and are more effective at labeling rare events in whole blood than micron sized particles [17]. A COMSOL simulation showed that magnetic field gradients on the order of ΔB = 300 T/m can be reproducibly generated across the 800 µm flow cell chamber (Figure S3). The combined effects of a threshold for volume magnetic saturation and sheath layer separation from the trapping region results in highly selective trapping of target cells and minimal nonspecific capture.

To evaluate whether this magnetophoretic separation was sufficient to recover target cells, a series of control samples were processed to examine the efficiency and purity of target cell recovery from whole blood using epithelial cell adhesion molecule (EpCAM) specific antibodies. Three tumor cell lines, MCF7, HCC1419 and A549, were selected because they expressed incremental numbers of EpCAM receptors. Flow cytometry confirmed that the relative expression of EpCAM varies over a 30-fold range (Figure 1C). Tumor cell lines were spiked into NHD blood at 10, 50 or 100 cells per mL. EpCAM expressing cells were recovered from 2 mL samples using the CTC flow cell (Figure 1C). For intermediate and high levels of expression of EpCAM, as on HCC1419 and MCF7 cells, the antigen density was sufficient to recover target cells with an 98% average efficiency over the tested range of input densities. A549 cells, which express 30-fold less EpCAM than MCF7, were recovered with 19%+/−9% efficiency. Thus, saturation magnetization of a particle remains a variable established by the number of receptors available for ferrofluid binding.

**Figure 2:**
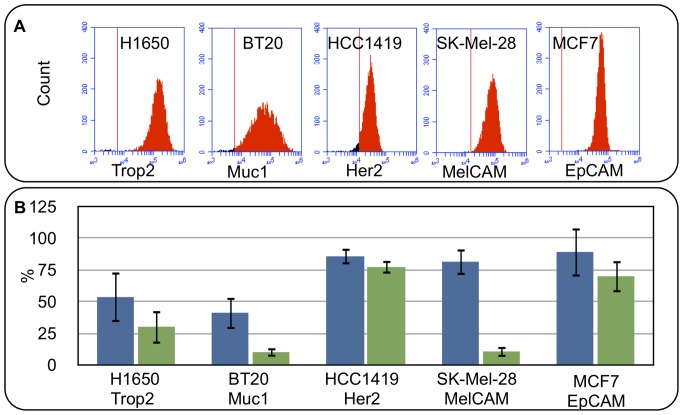
CTC Flow cell operation and performance. (A) Flow cytometry histograms of H1650, BT20, H1419, SK-Mel-28 and MCF7 cells labeled with 1 ug/mL of anti Trop2, Muc1, Her2, MelCAM and EpCAM antibodies respectively. Control antibody threshold is indicated by the red line (B) Recovery and purity of target cells using magnetophoretic capture from NHD blood: 90 cells/mL were spiked into NHD blood and purified on the LiquidBiopsy platform. Recovered cells were enumerated in the flow cell. Graphs show average recovery and purity of target cells +/−1 SD from 4 experiments for H1650, BT20, H1419 and SK-Mel-28 capture and 100 experiments for MCF7 capture.

While EpCAM is the most commonly chosen antibody for CTC capture, it is not highly expressed on all epithelial cancers and can not be used to recover cells from non epithelial cancers such as melanoma. To enable versatility, this platform uses a strepavidin/biotin linker system to attach the capture antibody to the magnetic particles. Therefore, any biotinylated reagent can be used to capture fluid phase targets. Four tumor cell lines, H1650, BT20, HCC1419 and SK-Mel-28, were selected because they expressed alternative cancer associated surface markers including Trop2, Muc1, Her2 and MelCAM. Flow cytometry confirmed the specific binding of each antibody to its target cell line (Figure 2A). These tumor cell lines were then spiked into NHD blood at a concentration of 90 cells per mL. Target cells were recovered from 8 mL samples using the LiquidBiopsy platform.

Target cell recovery and purity are reported in Figure 2B. The antibodies varied in recovery efficiency and sample purity. MelCAM, for instance, showed 82%+/−10% recovery of SK-Mel-28 cells but a low purity of 11%+/−4%. The low purity was attributed to specific capture of memory effector lymphocytes which are reported to express MelCAM [18]. In contrast the Her2 antibody demonstrated 86%+/−6% recovery of HCC1419 cells and sample purity of 77%+/−5%. This is comparable to EpCAM capture of MCF7 cells for which the average recovery of 100 tests was 89%+/−19% and the average purity was 70%+/−13%.

### LiquidBiopsy™ Platform Design and Performance

**Figure 3:**
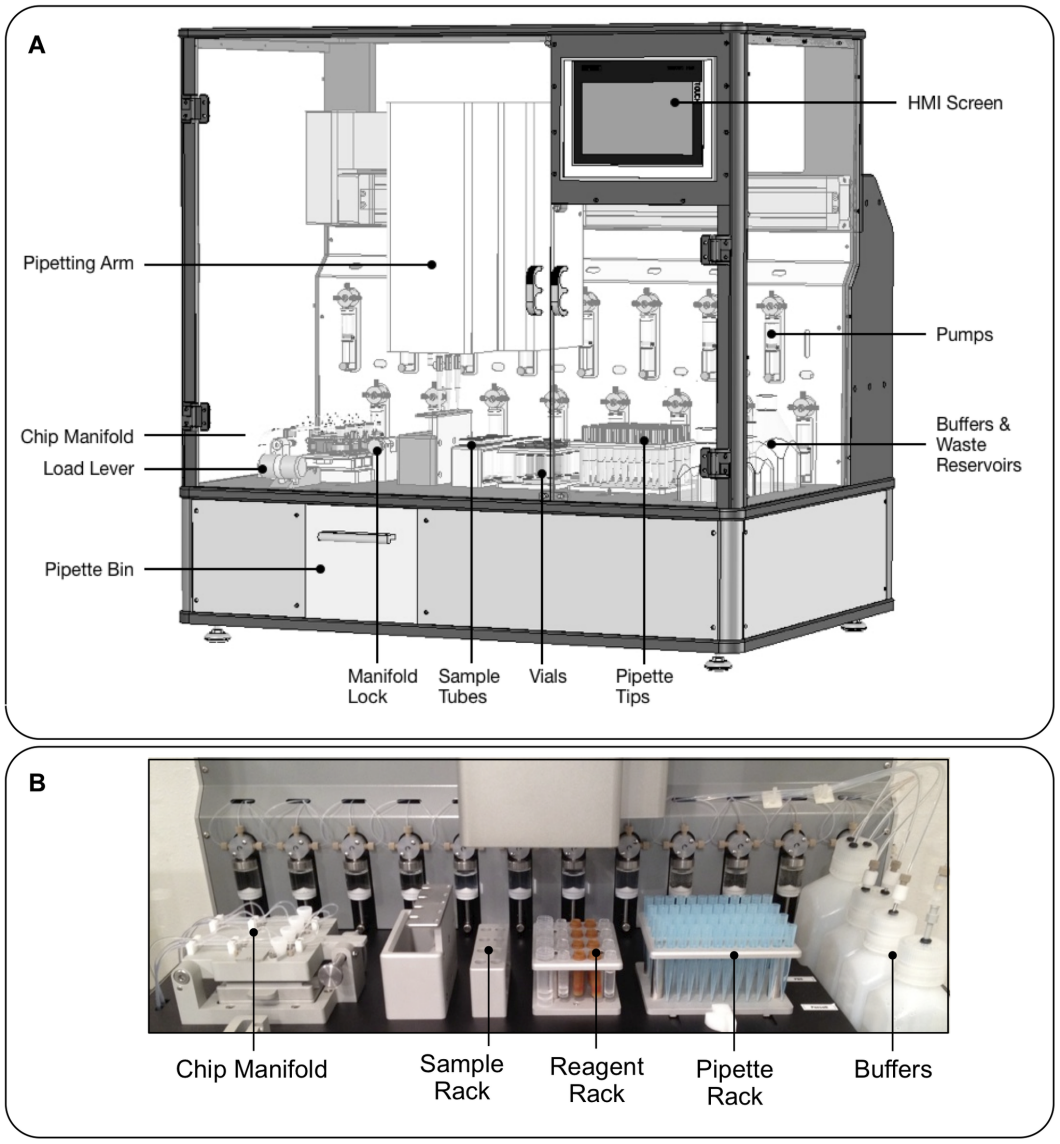
LiquidBiopsy automated platform. (A) Diagram of the LiquidBiopsy platform. (B) Closeup of the platform worksurface. A X-Y pipetting arm with 4 pipettor heads transfers sample, buffers and antibody stains into 4 flow cells in the manifold. Sheath buffers are controlled by pumps on the rear wall of the platform.

Clinical sample handling requires a recovery process that can be validated and can accommodate larger volumes and throughput. To this end, a platform was designed that would automate the handling of the CTC flow cell, buffer requirements and staining protocols (Figure 3). The LiquidBiopsy platform replicates the manual protocol with a few necessary changes. 1)The bottom buffer was switched from a sucrose base to an Iohexol base. This eliminated the need for narrow temperature control in order to maintain optimal density and flow rates, enabling room temperature operation. 2) Sample volume was increased to 7.5 mL. 3) The original cytokeratin 8 specific reagent CAM 5.2 was replaced with the pan cytokeratin C11 antibody to allow detection of a broad range of cytokeratins.

With the production of several automated platforms it became possible to execute a rigorous validation to determine the performance characteristics of the LiquidBiopsy platform as a tool for isolation and enumeration of CTCs present in peripheral blood samples. For the purpose of this validation CTCs were defined as EpCAM+, CK+, CD45− cells with a defined nucleus. The cell lines used in the validation were MCF7 (breast adenocarcinoma) and H1650 (lung adenocarcinoma). These two cell lines were selected because they express high levels of EpCAM and a range of cytokeratins enabling us to determine the platform's peak performance.

Baseline performance characteristics for the LiquidBiopsy platform were measured by adding MCF7 or H1650 cells into NHD blood at incremental concentrations in triplicate from 3 to 900 cells/mL and measuring the percent error, a measure of accuracy, and percent coefficient of variation (% CV), a measure of precision, at each input (Table 1). This range was selected because it exceeded the observed range in historically tested cancer patient samples. For MCF7 cells, % error was less than 30% between the range of 9–90 cells/mL; however, % CV was higher than 30% for all concentrations except 90 cells/mL. The high % error and % CV are due to a range of factors including a 10% variation in spike in concentration and enumeration errors from MCF7 cell clustering.

**Table 1:** Measurements of accuracy and precision of LiquidBiopsy capture of MCF7 and H1650 cells.

Expected Cell Density(CTCs/mL)	Accuracy (% error)		Precision (% CV)	
	MCF7	H1650	MCF7	H1650
3	51.8	n/a	36.4	n/a
9	29.5	20.0	36.0	19.9
30	29.2	12.5	40.8	24.4
90	24.6	19.5	9.1	13.3
300	45.4	30.6	29.9	11.1
900	47.9	44.3	45.1	17.9

Enumeration errors introduced from MCF7 clustering were investigated by testing the more monodispersed H1650 cell line. Due to the high variation in adding only 3 cells/mL, from stochastic sampling error [19], this concentration was excluded from the second test. There was a measurable improvement in both accuracy and precision when using H1650 cells with % error dropping to 20% or less between the range of 9–90 cells/mL and % CV decreasing to less than 25% for all concentrations tested (Table 1). These results support the contention that the tendency of target cells to cluster has a significant effect on the platform's accuracy and precision.

**Figure 4:**
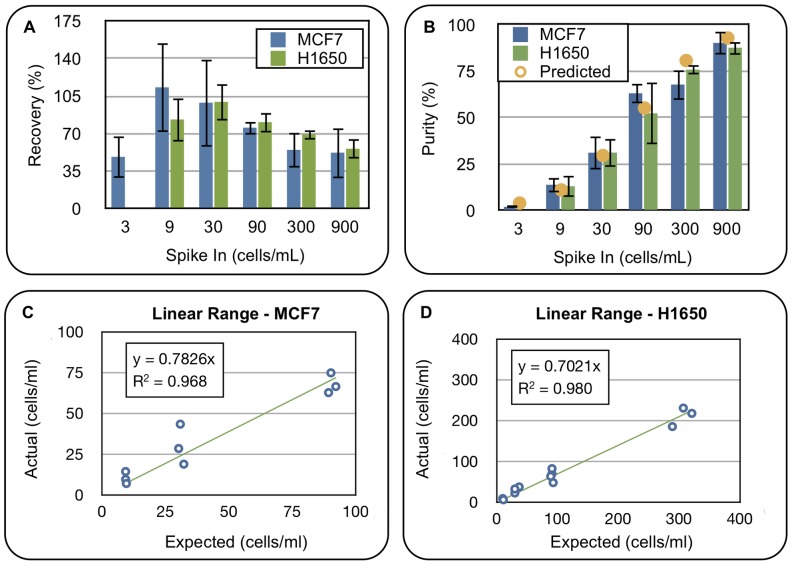
Validation of Automated CTC flow cell operation on LiquidBiopsy platform. (A) Plot of CTC recovery as a function of spike-in density for MCF7 and H1650 cells. (B) Plot of sample purity as a function of spike in density for MCF7 and H1650 cells. The orange dots indicate the predicted purity of MCF7 cells if the non target recovery is held constant at 55 cells/mL. (C) Raw data and linear fit to MCF7 recovery curve from 9 to 90 cells/mL. (D) Raw data and linear fit to H1650 recovery curve from 9 to 300 cells/mL.

Two critical operational parameters for this CTC assay are recovery and purity. High recovery, >70%, ensures that cells of interest will be recovered from clinical samples. High purity, >10%, enables unrestricted molecular access to the recovered CTC population. The test achieved greater than 70% recovery for samples spiked with 9–90 MCF7 cells/mL and 9–300 H1650 cells/mL using the EpCAM capture antibody (Figure 4A). Carry over of nonspecific cells is independent of the CTC content within a sample and averages 55 cells/mL; therefore, sample purity is related to CTC concentration by the equation 100*[CTC]/([CTC]+55). Figure 4B confirms that this curve accurately predicts the observed purity for MCF7 and H1650 cells. To further confirm specificity of the EpCAM capture system, recovery of two EpCAM negative cell lines were tested on the platform at a spike in density of 10,000 cells/mL. Average recovery of both the Daudi, a B cell lymphoma, and the Jurkat, a T cell leukemia, cell line was less than 0.025%±0.3% (Table S2).

Another key performance characteristic for a quantitative assay is that there is a linear relationship between the amount of analyte added and the amount recovered. For each cell line, linearity was assessed over several data subsets to determine which range had the best linear fit. For MCF7 cells the range from 9–90 cells/mL is highly linear with an R^2^ of 0.968 and an average recovery of 78% as measured by the slope (Figure 4C). For H1650 cells, the range of 9–300 cells/mL has an R^2^ of 0.980 and an average recovery of 70% (Figure 4D).

To determine if the drop in percent recovery observed for both cell lines at high concentrations was due to either saturation of recovery sites on the flow cell or insufficient capture antibody, NHD blood was spiked with 1×10^6^ HCC1419 breast cancer tumor cells and recovered in the CTC flow cell. The result was target cells densely recovered throughout the capture region of the flow cell (Figure S4). The cell density was so high that they could not be effectively enumerated but immunofluorescence confirmed that they were predominately HCC1419 cells. While this experiment does not prove that the LiquidBiopsy platform can capture all 1×10^6^ cells, it does demonstrate that the capture and detection reagents are not limiting in the region of 900 cells/mL.

A more probable cause for the drop in recovery is undercounting during enumeration at high concentrations. For the sake of expediency our scanner only images in one plane and may miss events in 3D clusters. This is consistent with the observation that recovery of the more monodispersed H1650 cells decreases at a slower rate than the clustering MCF7 cells.

With the linear range of MCF7 recovery determined to be from 9–90 cells/mL, the next step was to establish the intra-assay and inter-assay precision. The intra-assay precision was measured at 23% and 20% for samples spiked with 9 or 90 cells/mL, respectively. At least 10% of this variation at both concentrations can be attributed to the spike in process. The contribution to variation from the spike in process is likely higher at the lower concentration due to stochoastic sampling. The remaining variation can be attributed primarily to operator technique, platform performance and enumeration errors.

For clinical implementation it is critical that the LiquidBiopsy platform be robust to operational parameters such as run day, operator or platform; therefore, inter-assay precision was evaluated using the study design outlined in Table S1. No factor contributed more than 15% variation to the overall measurement and the variation attributed to run day and platform dropped to 5% and below for a density of 90 cells/mL (Table 2). A factorial ANOVA with an alpha level of 0.05 determined that the only statistically significant factor affecting variance was initial spike in density (Table S3).

**Table 2:** Results of intra and inter assay precision studies.

	Precision (% cv)			
Expected Cell Density(CTCs/mL)	Intra-assay	Inter-assay (Day)	Inter-assay (Operator)	Inter-assay (Platform)
9	23.3	14.8	13.2	15.3
90	19.5	5.0	10.1	1.8

The final step before molecular analysis is recovery of the cells from the flow cell into a PCR tube. The cells are recovered using the custom SpinElute tube that is shown in Figure S5. Cell recovery was evaluated both by PCR and imaging (Figure S5B and S5C). For the PCR test, 500 WBCs were directly added to a PCR tube or injected into and recovered from a flow cell. The quantity of genomic DNA released was assessed using a TaqMan probe for chromosome 9p. Figure S5B confirms that the same amount of genomic DNA was recovered from cells pipetted directly into the PCR tube compared with cells which were recovered from the flow cell. As the cells used in the PCR assay had not undergone the whole LiquidBiopsy process, a second elution evaluation was executed using an imaging readout. NHD samples were spiked with 30 MCF7 cells/mL and processed using the LiquidBiopsy platform. The flow cells were imaged before and after elution to determine if all the cells had been recovered. 100% of the MCF7 cells and an average of 99.5% of the donor derived cells were recovered from the 4 tested flow cells (Figure S5C).

### Molecular Access to CTC

A key differentiator of the LiquidBiopsy platform compared to other approaches is the ability to recover a highly purified sample which is suitable for subsequent standard molecular analysis. This is possible because of our high recovery of target cells and low recovery of non target cells. As demonstrated in Figure 4B the non target recovery is independent of target density and averages 55 cells/mL. The validation data predicts that only 6 CTC/mL need to be recovered to achieve a purity of 10% and less than 1 CTC/mL to achieve a purity better than 1%.

**Figure 5:**
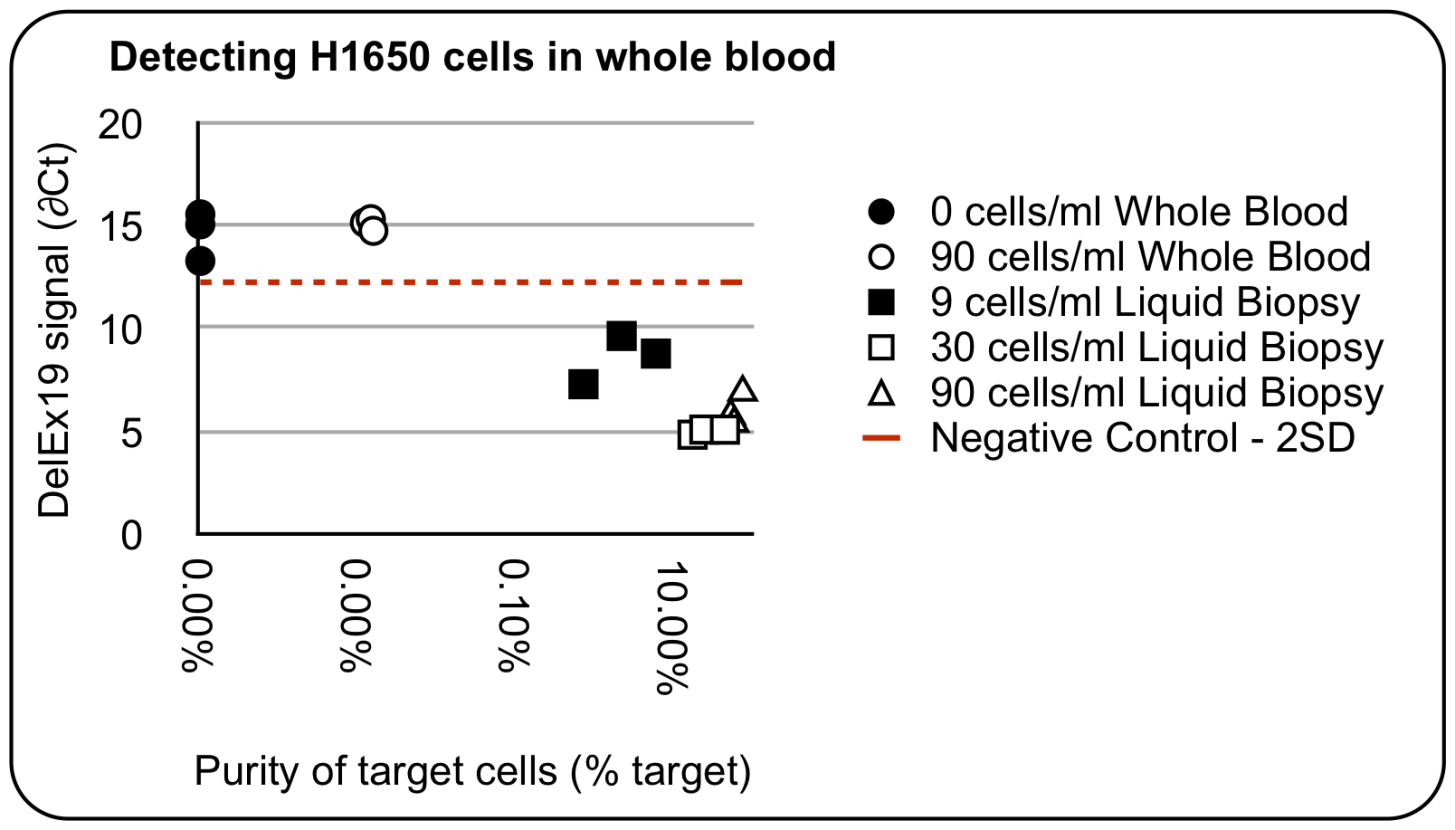
Direct molecular detection of tumor cells recovered from blood. Results of a TaqMan PCR mutation assay for the EGFR Exon 19 deletion mutation in H1650 cells. DNA recovered from incremental numbers of H1650 tumor cells spiked into whole blood and processed on the LiquidBiopsy platform are indicated. The mutation is undetectable in whole blood even at the highest input.

To evaluate the importance of this partition efficiency, the ability to detect an EGFR mutation in the H1650 cell line recovered from whole blood was evaluated. Captured cells were recovered from the CTC flow cell by centrifugation and whole genome amplified to produce sufficient template for PCR. A Taqman assay was performed evaluating the template for the presence of control wildtype EGFR template and the EGFR Ex19-del mutation. Incremental numbers of H1650 cells could be detected at all spike in levels using standard Taqman PCR (Figure 5). The Exon 19 deletion could be detected when as few as 9 cells/mL were spiked into NHD blood with a decreasing ∂Ct as more cells were added to the blood sample. The mutation was not detected within 40 PCR cycles in the RBC depleted blood even at the highest input concentration. Thus tumor cells can be quantitatively enriched sufficiently by the LiquidBiopsy platform to allow direct detection of mutant DNA. This average carryover of 55 non target cells/mL can support a number of standard molecular analysis techniques including PCR and next generation sequencing based approaches.

## Discussion

The LiquidBiopsy platform is a high throughput system which can isolate and purify circulating tumor cells from whole blood with minimal processing. The LiquidBiopsy flow cell can be analyzed on a standard microscope and the cells can be easily recovered for downstream analysis. The platform recovers an average 70% of monodispersed H1650 cells with a % error less than 20% and a % CV less than 25% over the range of 9–300 spiked cells/mL in NHD blood. For the clustering MCF7 cells, the platform recovered an average 78% with a % error of less than 30% over the range of 9–90 spiked cells/mL. Recovery of both cell lines was highly linear across the aforementioned range. An inter-assay study demonstrated that there is no significant effect of run day, operator or platform on recovery indicating that the platform is ready for deployment at multiple sites.

For the purpose of validating the platform, CTCs were recovered using EpCAM and visually confirmed as CK+ and CD45− using immunofluorescence. This is the “legacy” definition of a CTC inherited from validation of the CellSearch platform which demonstrated a prognostic link between clinical outcomes and CTC numbers using this definition [9], [10]. One problem with this definition is that EpCAM and CK are broadly expressed on both healthy and cancerous epithelial cells. The assumption is that since epithelial cells are not a normal component of healthy blood, any cells expressing epithelial markers must be tumor derived; however, elevated numbers of EpCAM+/CK+/CD45− cells have been found in the blood of healthy donors and patients with benign disease [14], [20]. Therefore, while counting these events may be prognostically associated with outcome, enumeration alone reveals little about the nature of the individual's cancer or the appropriate treatment approach.

Numerous methods have been utilized for the recovery of circulating tumor cells from whole blood with variable success. Bulk depletions or antigen independent mechanisms have yielded poorly defined and inefficiently isolated populations [21]–[23]. Microfluidic approaches have offered certain advantages but examples to date have been limited by low throughput and high non target carry over, or have been research platforms that lack the automation and validation necessary to deploy in a clinical lab setting [24]. Critically, no platform has been able to consistently process sufficient volumes, in a reasonable time, with sufficient purity to enable molecular analysis.

A key feature of the LiquidBiopsy platform is the ability to process relatively large volumes of whole blood, while recovering whole CTCs in a PCR tube at purities consistently greater than 1%. The average non target recovery by the LiquidBiopsy platform from a 7.5 mL whole blood sample is 55 cells/mL. Most CTC purification systems do not report purity numbers but of those that do “high” purity is described as 500–1500 leukocytes/mL [22], [25]. The LiquidBiopsy's 10 fold improvement in sample purity over the best reported numbers from other methods makes cell pellets from the LiquidBiopsy platform uniquely viable for downstream analysis such as next generation sequencing.

To be useful, in addition to being of sufficient purity and quantity for molecular analysis, the CTC-derived molecular templates must represent an informative population. For this reason, although EpCAM is currently the marker of choice for immuno-based CTC purification platforms, there is concern that clinically important populations are missed [26]. One possible mechanism to explain why these important cell populations are missed is due to the fact that EpCAM expression is lost in cancer cells during the epithelial to mesenchymal transition [27], [28]. Other mechanisms may be possible as well, but regardless, it is an essential capability to use more than the EpCAM marker system for CTC recovery. These concerns have been addressed by the LiquidBiopsy platform. Consequently it is possible with this platform to recover non-epithelial cancers, as well as cells of epithelial origin which have lost epithelial characteristics.

The LiquidBiopsy platform is compatible with any biotinylated antibody or molecule. In this paper the feasibility for employing virtually any marker system with essentially no retooling, recalibration, or reformulation was demonstrated by recovering cells using 4 alternative antibodies: Her2, MelCAM, Muc1 and Trop2. With this platform, there is no rigid adherence to the EpCAM marker system.

The challenge of defining and understanding the biology of the CTC underpins the current, hotly debated question concerning the ontology and phylogeny of primary tumors and the development of metastases. These questions will only be settled with the molecular analysis and molecular descriptions of the cells termed CTCs. Tumor cells in circulation represent the most poorly studied population of cells in the cancer life cycle. Progress towards understanding the molecular complexity of this disseminated cancer population is now possible due to advances in cell isolation technology, such as the LiquidBiopsy platform.

## Supporting Information

Figure S1 **Effect of sheath from on red blood cell adhesion.** 20× bright field image of the flow cell surface with (A) and without (B) sheath flow.
(TIFF)

Figure S2 **Examination of sheath flow using buffers labeled with red or green fluorescent dyes.** (A) Diagram of CTC flow cell. Green dye is used in the top and bottom buffer layers and red dye is used in the sample layer. (B) Diagram indicating two positions imaged during analysis. At standard flow rates of 3 mL/hr (top), 5 mL/hr (middle) and 5 mL/hr (bottom), laminar flow is maintained for residence times above 100 s. The dye diffusion length is about 250 microns so the smearing at position 2 is expected but is not observed in cellular samples.
(TIFF)

Figure S3 **Simulation of magnetic force on cells.** In this figure, DeltB = |ΔB|(A) surface plot of ΔB. From low to high, color changes from blue (0) to red (1000 T/m). White areas are >1000 T/m. (B) ΔB across the middle of the middle of the microchannel in the CTC flow cell (assuming a channel depth of 0.8 mm). Modeled with 200 µm (red line) or 500 µm (blue line) actual coverslip thickness.
(TIFF)

Figure S4 **Capture of 1×10^6^ HCC1419 cells.** 20× images of bright field, DAPI (DNA) and FITC (Cytokeratin) at two positions on a flow cell which processed blood spiked with 1×10^6^ cancer cells/mL.
(TIFF)

Figure S5 **SpinElute tube successfully recovers cells for downstream analysis.** (A) SpinElute tube with flow cell inserted and PCR tube attached. (B) Results of a TaqMan PCR probe for chromosome 9p. The graph indicates the threshold cycle for detection of the Chr:9p probe in triplicate determinations for 4 control replicas and 16 test elutions. The red box indicates the average threshold cycle for the 4 controls+2 SD. (C) Target and non target cell recovery from flow cell assessed by image analysis before and after elution.
(TIFF)

Table S1 **Design of the Inter-Assay Study.**
(DOC)

Table S2 **Results of testing EpCAM- cells on the platform.**
(DOC)

Table S3 **Results of Factorial ANOVA on Inter-Assay Study Data.**
(DOC)
