# Supplementary figures and images for: Circulating Tumor Cells: Clinically Relevant Molecular Access Based on a Novel CTC Flow Cell
Source: PLoS One. 2014 Jan 29;9(1):e86717. doi: 10.1371/journal.pone.0086717 (PMC3906064; doi:10.1371/journal.pone.0086717)

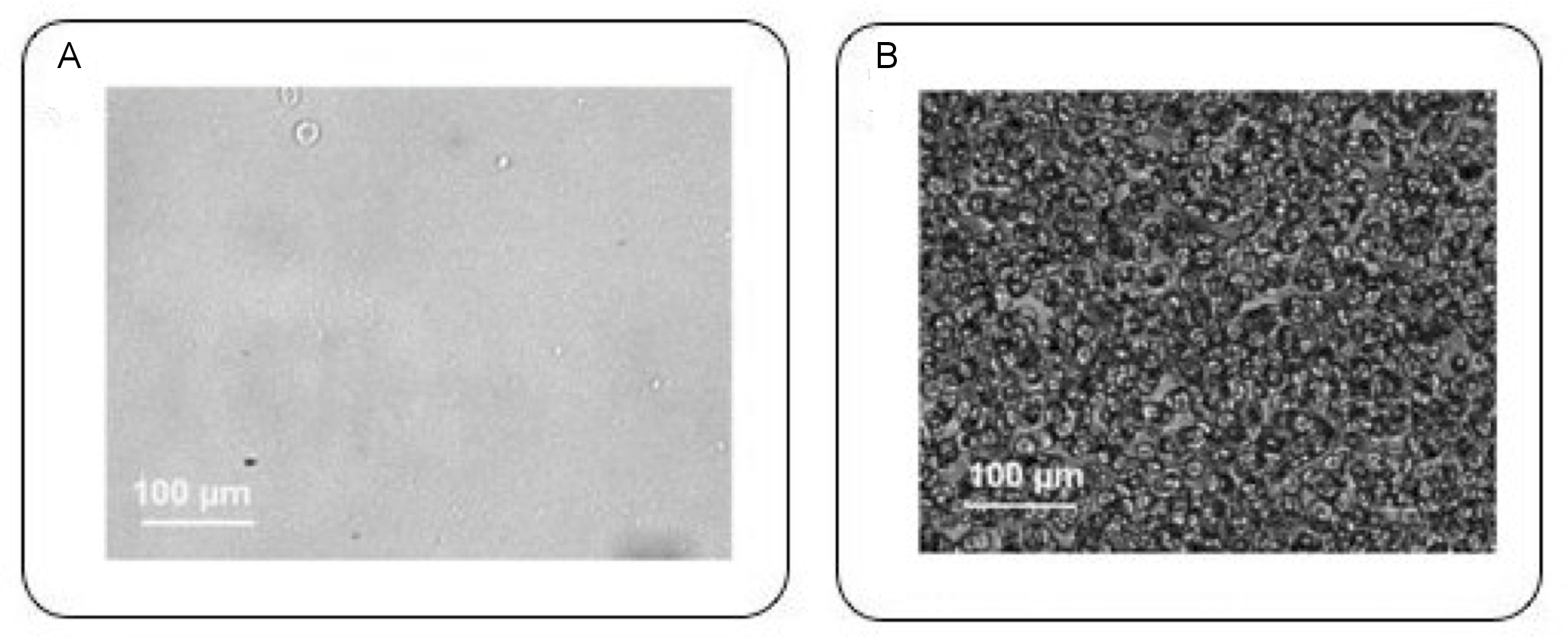

Supplement: Figure S1 — Effect of sheath from on red blood cell adhesion. 20× bright field image of the flow cell surface with (A) and without (B) sheath flow. (TIFF) [file pone.0086717.s001.tiff]

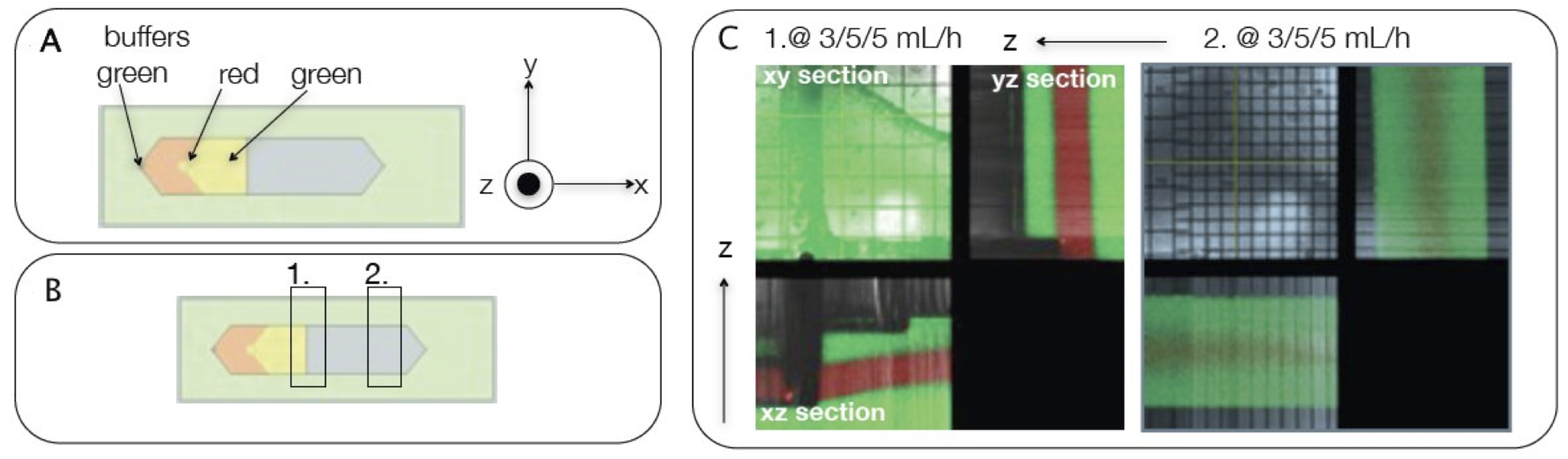

Supplement: Figure S2 — Examination of sheath flow using buffers labeled with red or green fluorescent dyes. (A) Diagram of CTC flow cell. Green dye is used in the top and bottom buffer layers and red dye is used in the sample layer. (B) Diagram indicating two positions imaged during analysis. At standard flow rates of 3 mL/hr (top), 5 mL/hr (middle) and 5 mL/hr (bottom), laminar flow is maintained for residence times above 100 s. The dye diffusion length is about 250 microns so the smearing at position 2 is expected but is not observed in cellular samples. (TIFF) [file pone.0086717.s002.tiff]

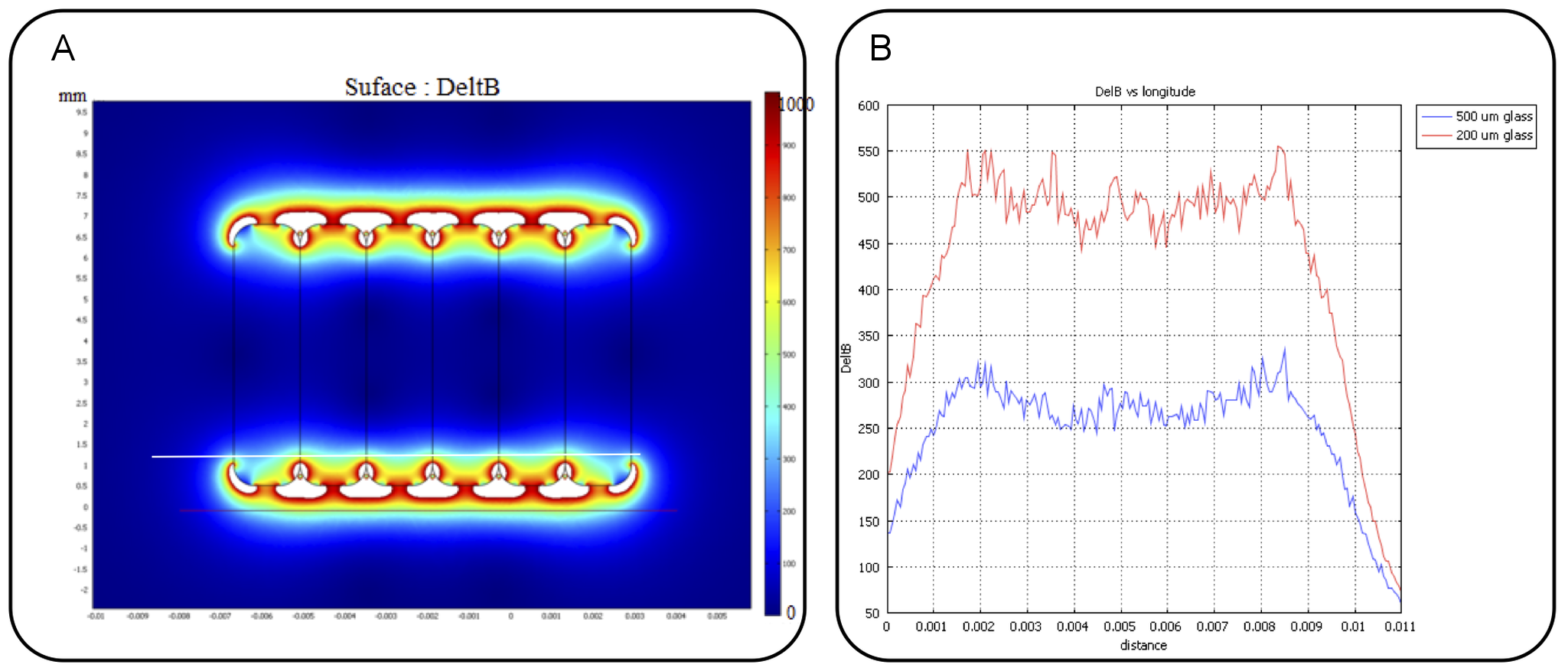

Supplement: Figure S3 — Simulation of magnetic force on cells. In this figure, DeltB = |ΔB|(A) surface plot of ΔB. From low to high, color changes from blue (0) to red (1000 T/m). White areas are >1000 T/m. (B) ΔB across the middle of the middle of the microchannel in the CTC flow cell (assuming a channel depth of 0.8 mm). Modeled with 200 µm (red line) or 500 µm (blue line) actual coverslip thickness. (TIFF) [file pone.0086717.s003.tiff]

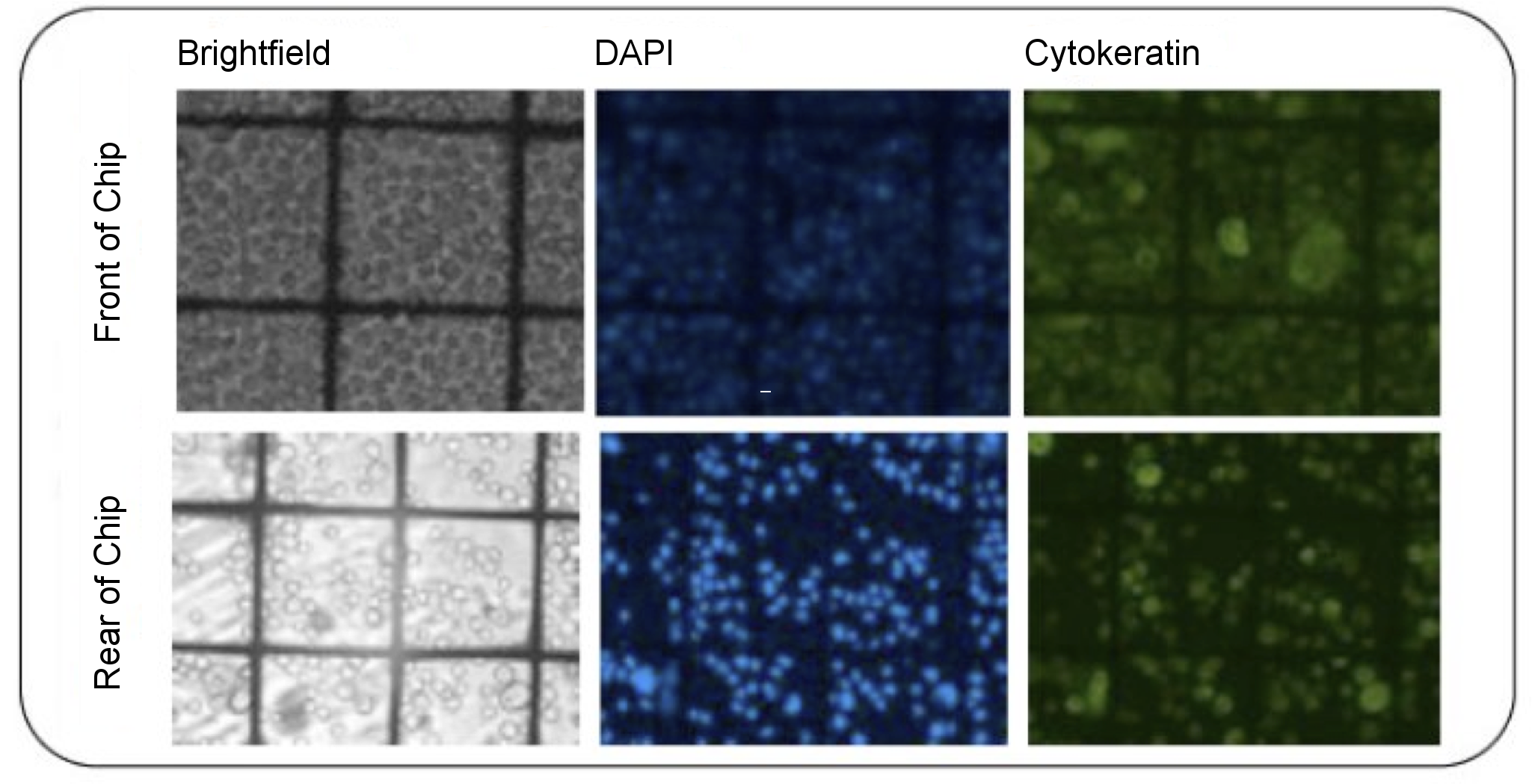

Supplement: Figure S4 — Capture of 1×10^6^ HCC1419 cells. 20× images of bright field, DAPI (DNA) and FITC (Cytokeratin) at two positions on a flow cell which processed blood spiked with 1×10^6^ cancer cells/mL. (TIFF) [file pone.0086717.s004.tiff]

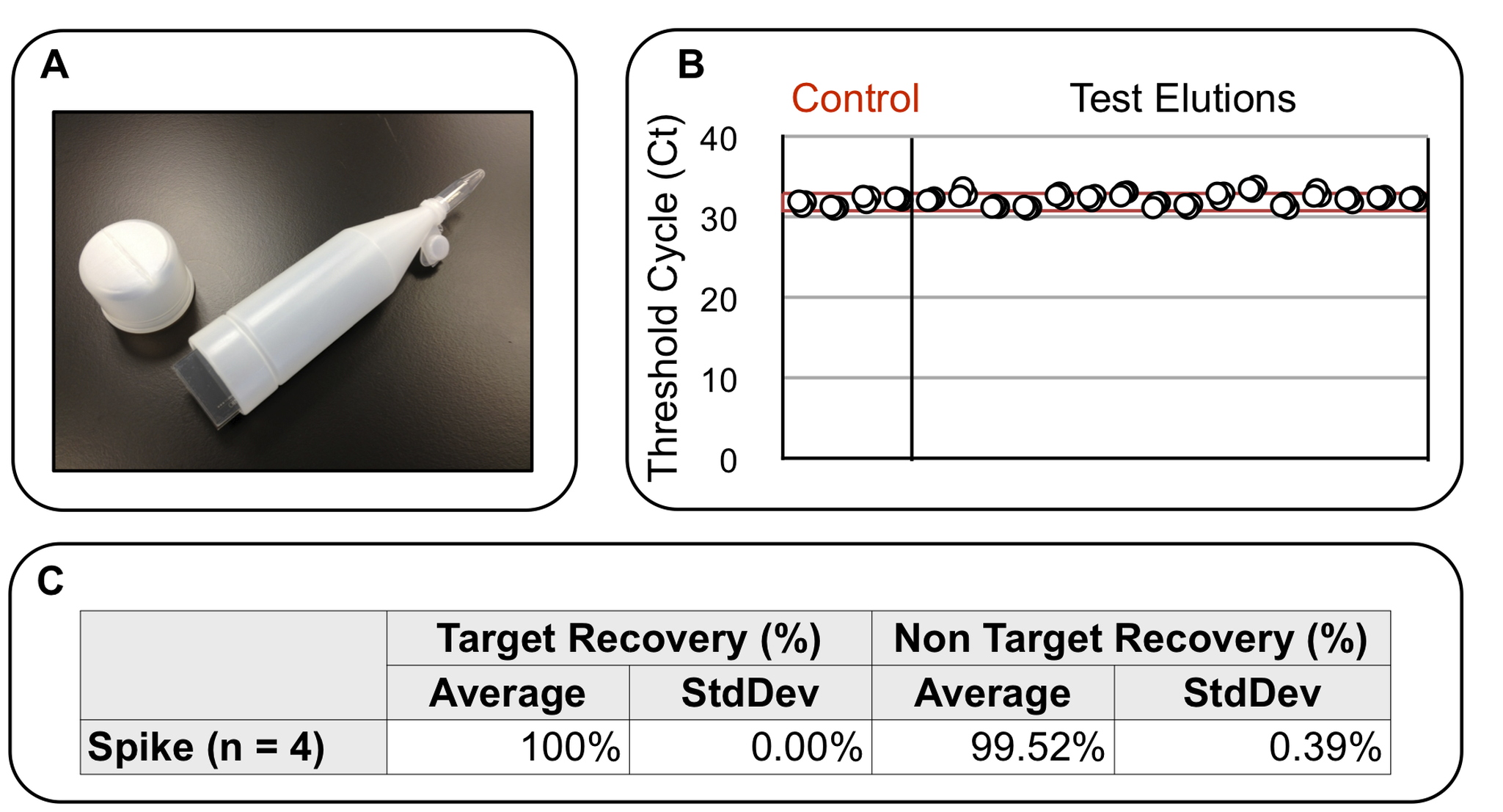

Supplement: Figure S5 — SpinElute tube successfully recovers cells for downstream analysis. (A) SpinElute tube with flow cell inserted and PCR tube attached. (B) Results of a TaqMan PCR probe for chromosome 9p. The graph indicates the threshold cycle for detection of the Chr:9p probe in triplicate determinations for 4 control replicas and 16 test elutions. The red box indicates the average threshold cycle for the 4 controls+2 SD. (C) Target and non target cell recovery from flow cell assessed by image analysis before and after elution. (TIFF) [file pone.0086717.s005.tiff]
