# Supplementary material for: Circulating Tumor Cells: Clinically Relevant Molecular Access Based on a Novel CTC Flow Cell
Source: PLoS One. 2014 Jan 29;9(1):e86717. doi: 10.1371/journal.pone.0086717 (PMC3906064; doi:10.1371/journal.pone.0086717)
Supplement: Table S1 — Design of the Inter-Assay Study. (DOC) [file pone.0086717.s006.doc]

| **Expected Cell Density**  **(CTCs/mL)** | **Operator** | **LB Platform** | **Repeats** | **ID** |
| --- | --- | --- | --- | --- |
| 9 | A | A | 5 days | AA |
|  | A | B | 5 days | AB |
|  | B | A | 5 days | BA |
|  | B | B | 5 days | BB |
| 90 | A | A | 5 days | AA |
|  | A | B | 5 days | AB |
|  | B | A | 5 days | BA |
|  | B | B | 5 days | BB |
