# Supplementary material for: Circulating Tumor Cells: Clinically Relevant Molecular Access Based on a Novel CTC Flow Cell
Source: PLoS One. 2014 Jan 29;9(1):e86717. doi: 10.1371/journal.pone.0086717 (PMC3906064; doi:10.1371/journal.pone.0086717)
Supplement: Table S2 — Results of testing EpCAM- cells on the platform. (DOC) [file pone.0086717.s007.doc]

|  | **Recovery (%)** | |
| --- | --- | --- |
|  | **Average** | **StdDev** |
| **Daudi (n=8)** | 0.02% | 0.03% |
| **Jurkat (n=8)** | 0.01% | 0.02% |
