# Supplementary material for: Circulating Tumor Cells: Clinically Relevant Molecular Access Based on a Novel CTC Flow Cell
Source: PLoS One. 2014 Jan 29;9(1):e86717. doi: 10.1371/journal.pone.0086717 (PMC3906064; doi:10.1371/journal.pone.0086717)
Supplement: Table S3 — Results of Factorial ANOVA on Inter-Assay Study Data. (DOC) [file pone.0086717.s008.doc]

|  | **DF** | **SS** | **MS** | **F** | **P-value** |
| --- | --- | --- | --- | --- | --- |
| **CONSTANT** | 1 | 73274 | 73274 | 308.895 | < 1e-08 |
| **Spike** | 1 | 24206 | 24206 | 102.046 | < 1e-08 |
| **Day** | 1 | 16.2 | 16.2 | 0.068 | 0.7954 |
| **Operator** | 1 | 422.5 | 422.5 | 1.781 | 0.1909 |
| **Platform** | 1 | 67.6 | 67.6 | 0.285 | 0.5969 |
| **Operator.Platform** | 1 | 902.5 | 902.5 | 3.805 | 0.0594 |
| **ERROR1** | 34 | 8065.2 | 237.21 |  |  |
